# Methicillin-Resistance in *Staphylococcus aureus* Is Not Affected by the Overexpression in Trans of the *mecA* Gene Repressor: A Surprising Observation

**DOI:** 10.1371/journal.pone.0023287

**Published:** 2011-08-02

**Authors:** Duarte C. Oliveira, Hermínia de Lencastre

**Affiliations:** 1 Laboratory of Molecular Genetics, Instituto de Tecnologia Química e Biológica, Universidade Nova de Lisboa, Oeiras, Portugal; 2 Laboratory of Microbiology, The Rockefeller University, New York, New York, United States of America; Universite Libre de Bruxelles, Belgium

## Abstract

Methicillin-resistant *Staphylococcus aureus* (MRSA) is intrinsically cross-resistant to virtually all β-lactam antibiotics. The central determinant for the MRSA phenotype is the *mecA* gene, whose transcriptional control may be mediated by a repressor (*mecI*) and a sensor/inducer (*mecR1*). The *mecI-mecR1-*mediated induction of *mecA* takes several hours rendering the strains phenotypically susceptible in spite of the presence of the resistance gene. Therefore, it has been proposed that the full resistance to β-lactams observed in many contemporary clinical MRSA strains requires a non-functional *mecI-mecR1* regulatory system. The *mecA* gene is embedded in a large chromosomal cassette (the SCC*mec* element) for which several structural types have been described. Some epidemic MRSA clones, typically expressing full β-lactam resistance, carry SCC*mec* elements that contain an intact *mecI-mecR1 locus* (e.g. SCC*mec* types II and III). We have addressed this apparent contradiction by first sequencing the *mecI* coding region and *mecA* promoter sequences in a collection of prototype MRSA strains characterized by different SCC*mec* types. A conserved non-sense mutation within *mecI* was detected in all SCC*mec* type III strains tested, presumably responsible for a non-functional truncated MecI protein and, therefore, explaining the full resistance phenotype. In SCC*mec* type II strains no conserved mutations were found. We next transformed a collection of prototype MRSA epidemic strains with a recombinant plasmid overexpressing a wild-type copy of *mecI*. Surprisingly, for the great majority of the strains no significant alterations in the phenotypic expression of β-lactam resistance could be detected. These findings were confirmed and further explored, challenging the currently accepted mechanism of *mecA* transcriptional control. Our observations suggest the existence of yet unidentified additional determinants involved in the transcriptional control of *mecA* gene and point to a revision of the *mecA* regulatory mechanism in contemporary MRSA strains.

## Introduction

Methicillin-resistant *Staphylococcus aureus* (MRSA) is a leading cause of nosocomial infections worldwide and has also emerged as a community-associated pathogen [1]. MRSA are inherently cross-resistant to virtually all β-lactam antibiotics, the most effective and widely used class of antimicrobials. Moreover, MRSA clinical strains are quite often multi-drug resistant, reducing significantly the therapeutic options for the treatment of staphylococcal infections.

The MRSA characteristic phenotype is due to the presence of *mecA*, which encodes a penicillin-binding protein (PBP), PBP2a, with reduced affinity for β-lactams [2], [3]. *mecA* is embedded in a large heterologous chromosomal cassette, the SCC*mec* element [4]. Some MRSA strains carry upstream to the *mecA* gene the regulatory genes *mecI-mecR1* encoding for a repressor and a sensor/inducer of the *mecA* expression, respectively [5]. This genetic organization is similar to the β-lactamase locus that encodes for penicillin-resistance only, and contains the structural gene (*blaZ*), a repressor (*blaI*) and a sensor/inducer (*blaR1*). Apart from the identical structural organization between *mec* and *bla* systems, there is also good homology between the inducers (61% identity at the amino acid level) and the β-lactam binding domain of the repressors (44% identity at the amino acid level). In fact, there is a cross-talk between both regulatory systems, as each one alone is able to control the transcription of *mecA* and *bla*Z [6], [7]. However, the two regulatory systems differ remarkably in the induction efficiency: the *blaI-blaR1* system induces *mecA* in a few minutes, whereas the *mecI-mecR1* system takes several hours [6], [7], [8]. Actually, the *mecI*-*mecR1* mediated induction of *mecA* expression is so slow that, in clinical terms, fully functional *mecI* and *mecR1* genes render the cell phenotypically susceptible in spite of the presence of *mecA* – the so-called “pre-MRSA” phenotype [5], [9]. In agreement with this observation, the *in vitro* deletion of the *mecI* gene has been shown to increase the resistance levels to β-lactams in staphylococci [5], [9], [10].

Based on those observations, it has been postulated that full resistance to β-lactams, characteristic of many contemporary MRSA clinical strains, implies a non-functional *mecI-mecR1* regulatory system [5], [9]. As a matter of fact, the absence of *mecI* or the accumulation of point mutations in the *mecI* coding sequence, *mecI* ribosomal binding site or *mecA* gene promoter, have been found in several MRSA strains [11], [12], [13], [14], [15]. Nevertheless, in spite of the negative effect of the presence of MecI on the phenotypic expression of resistance, there is no clear direct correlation between the cellular amounts of *mecA* transcript or PBP2a protein and the phenotypic level of resistance (i.e. the minimum inhibitory concentration, MIC, for the strain) [16], [17]. Finally, the existence of other unknown determinants involved in *mecA* regulation, for instance mediating the signal transduction between the activated MecR1 and the promoter bound MecI, has been postulated based on contradictory experimental observations [13], [15], [18] and critical structural data analysis [19], [20]. Early studies on the β-lactamase regulon in *S. aureus* by Cohen and Sweeney have also suggested the existence of the *blaR2* locus, unlinked to *blaI-blaR1* and able to mediate the constitutive expression of *blaZ* [21].

Currently, MRSA clones are defined not only based on the type of genetic lineage but also on the type of SCC*mec* element they carry, as the same lineage may be associated with several SCC*mec* types [22], [23]. Several structural types of the SCC*mec* element have been described differing in size and genetic content [24]. The genetic organization of the *mecA* vicinity (*mec* gene complex) is one of the key characteristics used to define SCC*mec* types. In *S. aureus* three major *mec* classes have been described based mainly on the presence of insertion sequences and intact or disrupted *mecI-mecR1* sequences: class A has intact sequences for *mecI-mecR1*, whereas classes B and C have no *mecI* and partially deleted *mecR1* due to the integration of insertion sequences in the regulatory region of the *mecA*. Considering the eight major SCC*mec* types described so far in *S. aureus*, the *mec* gene complex class A characterizes SCC*mec* types II, III and VIII; class B, SCC*mec* types I, IV and VI; and class C, SCC*mec* types V and VII. According to the current model of *mecA* transcriptional control [5], [9], it is tempting to interpret the disruption of the *mecI-mecR1* regulatory system in SCC*mec* types I and IV–VII, as a strategy to overcome the tight *mecA* repression mediated by *mecI-mecR1* system in MRSA strains. However, it seems that there is no clear correlation between resistance levels and *mecI-mecR1* functionality, as some strains lacking *mecI-mecR1* have a very low resistance level, whereas some strains with complete *mecI-mecR1* locus are highly resistant [14], [15], [25]. In addition, the epidemicity of MRSA strains does not correlate with the *mecI-mecR1* functionality as well. For example, two major pandemic nosocomial MRSA clones – the New York/Japan (or ST5-II) and the Brazilian (or ST239-III) clones – are characterized by SCC*mec* types II and III, respectively, and have a complete *mecI-mecR1* locus [22], [23].

In this study, we have addressed the puzzling lack of correlation between SCC*mec* type (i.e. *mec* gene class) and resistance phenotype in order to clarify how the *mecA* transcription is controlled in pandemic MRSA strains. We first characterized by DNA sequencing the *mecA* regulatory locus in prototype strains of SCC*mec* types II and III (i.e. with a complete *mecI-mecR1* locus). We found a conserved point mutation within the *mecI* coding-sequence among SCC*mec* type III strains, which introduces a premature stop codon resulting in truncated MecI repressor. Among the SCC*mec* type II strains we could not find any conserved sequence alteration either in the *mecI* coding sequence or in the *mecA* promoter that could suggest a non-functional *mecI-mecR1* system and, as such, justify the high-resistance levels to β-lactams. We thus decided to challenge the current model of *mecA* regulation by over-expressing *in trans* the wild-type MecI in a collection of prototype MRSA clinical strains. To our surprise, in most strains we could not detect any significant alterations in the phenotypic expression of oxacillin resistance. These observations suggest that other yet uncharacterized factors are involved in the control of the expression of β-lactam resistance in MRSA, namely by interfering with the *mecI*-mediated repression of *mecA*, and point to a revision of the current model for the transcriptional control of the resistance determinant in contemporary strains.

## Materials and Methods

### Bacterial strains and culture conditions

MRSA strains used in this study and their relevant characteristics are listed in Tables 1 and 2. All strains have been selected from large international collections and have been previously characterized in detail for genetic background by pulsed-field gel electrophoresis (PFGE), multi-locus sequence typing (MLST), and *spa* typing and also for SCC*mec* type. In Table 1, we have included prototype strains for three major pandemic MRSA lineages characterized by SCC*mec* types I–III [22]: the Iberian or ST247-I clone, the New York/Japan or ST5-II clone, and the Brazilian or ST239-III clone. In addition, in Table 1 we have also included two reference strains extensively used in studies addressing the β-lactam resistance mechanisms in *S. aureus*: strains COL [26] and N315 [9]. Strain COL is highly and homogenously resistant to oxacillin, has a non-functional *mecI-mecR1* system, it naturally lacks β-lactamase, and constitutively expresses *mecA* [26], [27]. Strain N315, has a very heterogeneous oxacillin-resistance phenotypic profile, has wild-type *mecI-mecR1* sequences, is β-lactamase positive and has an inducible expression of *mecA* [5], [9]. In Table 2, we list further MRSA strains that were selected to extended our initial collection in order to confirm the experimental observations. For this purpose, we have included more representative SCC*mec* type II strains and also strains characterized by SCC*mec* types IV–VI [28], [29], [30]. *S. aureus* strains were routinely grown overnight at 37°C under aerobic conditions on tryptic soy agar or tryptic soy broth (Difco, BD). *Escherichia coli* strains were grown in Luria-Bertani broth (Difco, BD) with aeration at 37°C. Chloramphenicol (20 µg/ml) and ampicillin (100 µg/ml) (Sigma-Aldrich) were used for selection and maintenance of *S. aureus* and *E. coli* transformants, respectively.

**Table 1:** Characteristics of the prototype MRSA strains used in this study.

				Relevant characteristics				Oxacillin MIC (µg/ml)e)	
Strain	Origin	Isolation date	Clonala) type	*mecI* b)	*mecR1* c)	P*mecA*	*bla* d)	Parental strain	Recombinant strainf)
COL	UK	1965	ST250 – I	neg.	IS::Δ*mecR1*	WT	neg.	>256	1.5
PER34	Spain	1989	ST247 – I	neg.	IS::Δ*mecR1*	WT	pos.	>256	>256
HPV107	Portugal	1992	ST247 – I	neg.	IS::Δ*mecR1*	WT	pos.	>256	>256
N315	Japan	1982	ST5 – II	WT	WT	WT	pos.	32	24
BK2464	USA	1996	ST5 – II	WT	WT	WT	pos.	>256	>256
HU25	Brazil	1993	ST239 – III	*mecI* *	WT	WT	pos.	>256	>256
BK2421	USA	1996	ST239 – III	*mecI* *	Δ*mecR1*	WT	pos.	>256	>256

Abbreviations: neg., negative; pos., positive; WT, wild-type.

Notes:

a) Clonal types as defined by MLST sequence type (ST) and SCC*mec* type.

b) neg. – negative (due to IS*1272* insertion); WT – wild-type sequence (N315);

*- mutated non-functional *mecI* at Gln_68_ (CAA→TAA);

c) IS::Δ*mecR1* – *mecR1* with no C-terminal sensor domain (due to IS*1272* insertion); Δ*mecR1* – 160 bp deletion in the C-terminal inducer domain.

d) The production of β-lactamase was assayed in induced and no induced cultures (see text for details). All strains positive for β-lactamase were inducible.

e) MIC as determined by E-test strips.

f) Parental strains transformed with a high copy number plasmid containing the wild-type *mecI* coding region (pGC2-*mecI* ^WT^).

**Table 2:** Characteristics of the extended collection of representative MRSA strains.

Strain	Origin	Isolation date	Clonala) type	Relevant characteristics				Oxacillin MIC (µg/ml)e)	
				*mecI* b)	*mecR1* c)	P*mecA*	β-lact.d)	Parental strain	Recombinant strainf)
USA100	USA	1995–2003	ST5-II	pos.	pos.	ND	pos.	64	64
USA200	USA	1995–2003	ST36-II	pos.	pos.	ND	pos.	>256	>256
HAR24	Finland	2002	ST36-II	pos.	pos.	ND	pos.	>256	>256
USA600	USA	1995–2003	ST45-II	pos.	pos.	ND	pos.	>256	>256
MW2	USA	1998	ST1-IV	neg.	IS::Δ*mecR1*	ND	pos.	32	32
HAR22	Finland	2002	ST22-IV	neg.	IS::Δ*mecR1*	ND	pos.	>256	>256
USA400	USA	1995–2003	ST1-IV	neg.	IS::Δ*mecR1*	ND	pos.	96	96
USA800	USA	1995–2003	ST5-IV	neg.	IS::Δ*mecR1*	ND	pos.	48	32
VNG17	Portugal	1992–1993	ST5-IV	neg.	IS::Δ*mecR1*	WT	neg.	16	0.25
RJP17	Portugal	1992–1993	ST5-IV	neg.	IS::Δ*mecR1*	WT	neg.	32	24
HSA49	Portugal	1993	ST5-IV	neg.	IS::Δ*mecR1*	ND	pos.	24	24
USA300	USA	1995–2003	ST8-IV	neg.	IS::Δ*mecR1*	ND	pos.	24	16
USA500	USA	1995–2003	ST8-IV	neg.	IS::Δ*mecR1*	ND	pos.	>256	>256
HAR38	Belgium	1995	ST45-IV	neg.	IS::Δ*mecR1*	ND	pos.	128	128
USA700	USA	1995–2003	ST72-IV	neg.	IS::Δ*mecR1*	ND	pos.	48	48
DEN2949	Denmark	2001	ST80-IV	neg.	IS::Δ*mecR1*	ND	pos.	64	64
WIS	Australia	1995	ST45-V	neg.	IS::Δ*mecR1*	ND	pos.	4	4
HDE288	Portugal	1996	ST5-VI	neg.	IS::Δ*mecR1*	ND	pos.	6	6

Abbreviations: neg., negative; pos., positive; WT, wild-type; ND, not determined.

Notes:

a) Clonal types as defined by MLST sequence type (ST) and SCC*mec* type.

b) neg. – negative (due to IS*1272* or IS*431* insertions); WT – wild-type sequence (N315); * - mutated non-functional *mecI*.

c) IS::Δ*mecR1* – *mecR1* with no C-terminal sensor domain (due to IS*1272* or IS431 insertions).

d) The production of β-lactamase was tested for induced and no induced cultures (see text for details). All strains positive for β-lactamase were inducible. Strains negative for the nitrocefin assay were tested for the presence of *blaZ*, blaI, and *bla*R1 by PCR.

e) MIC as determined by E-test strips.

f) Parental strains transformed with a high copy number plasmid containing the wild-type *mecI* coding region (pGC2-*mecI* ^WT^).

### DNA manipulations

DNA manipulations were performed by standard methods [31], [32]. Restriction enzymes were used as recommended by the manufacturer (New England Biolabs). Routine PCR amplification was performed with AmpliTaq DNA polymerase (Applied Biosystems) according to the manufacturer's recommendations. Wizard Plus Minipreps and Midipreps (Promega) purification systems were used for plasmid extraction. PCR and digestion products were purified with Wizard PCR Preps and Wizard DNA Clean-up systems (Promega). Ligation reactions were performed with the Rapid DNA Ligation Kit (Roche). DNA sequencing was performed by the Rockefeller University Protein/DNA Technology Center or by Macrogen (www.macrogen.com).

### β-lactamase detection

Detection of functional β-lactamase locus was performed either with BBL™ DrySlide™ Nitrocefin (BD) (strains listed in Table 1) or with nitrocefin disks (Sigma-Aldrich) (strains listed in Table 2) according to the manufacturer's recommendations for cultures grown overnight in TSB with and without induction with oxacillin at 0.5 µg/ml. Results were recorded after 30 min. incubation at room temperature. In the case of strains negative for the Nitrocefin assay, the absence of β-lactamase genes was confirmed by PCR with three pairs of primers targeting internal fragments of ca. 500 bp of *blaZ*, *bla*I, and *blaR1*. Primers were designed based on the available sequence at GenBank for Tn*552* of *S. aureus* (accession number: X52734); primer sequences were as follows (5′→3′): blaZ F, GAT AAG AGA TTT GCC TAT GC; blaZ R, GCA TAT GTT ATT GCT TGA CC; blaI F, GCA AGT TGA AAT ATC TAT GG; blaI R, GAA AGG ATC CAT TTT CTG TAC ACT CTC ATC; blaR1 F, CAT GAC AAT GAA GTA GAA GC; and blaR1 R, CTT ATG ATT CCA TGA CAT ACG.

### Phenotypic analysis

Initial susceptibility screening to oxacillin was determined for all parental and recombinant strains with 1 mg oxacillin diffusion disks prepared in-house [33]. For the MIC determination oxacillin E-test strips (AB Biodisk) were used for all strains. Overnight TSB cultures were adjusted to an optical density at 620 nm (OD_620_) of 0.08 (equivalent to 0.5 McFarland), plated onto Muller Hinton agar (Difco) plates supplemented with 2% NaCl, and incubated at 37°C for 24 h. Strains listed in Table 1 were also tested by population analysis profiles (PAPs), as previously described [34], [35]. In short, 10 µl drops of 10^0^, 10^−1^, 10^−2^, 10^−3^, 10^−4^, 10^−5^, and 10^−6^ dilutions of an overnight culture were plated on TSA plates containing 0, 0.75, 1.5, 3, 6, 12.5, 25, 50, 100, 200, 400, 800 µg/ml of oxacillin (Sigma). For each oxacillin concentration, colonies were counted for the first dilution with non-confluent growh after 24 h and 48 h of incubation at 37°C.

### DNA sequencing of *mecI* and the *mecA* promoter

Based on the sequence of the *mecA* regulatory region for the reference strain N315 (accession number D86934) two primer sets were designed for the amplification and sequencing of the *mecI* coding region and the promoter region of the *mecA* gene (P*mecA*). Primer sequences were as follows (5′→3′): mecI F, TTA CGC TTA CCG CTT TTTCG; mecI R, ATC AAG ACT TGC ATT CAG GC; PmecA F, GTA ACA GAT GAT TGT TGA CC; PmecA R, AAG ATG AAG TGG TAA TAG CG. DNA sequencing raw data analysis and multi-sequence alignments were performed using the DNA Star software package (Lasergene). All sequences have been deposited in GenBank with accession numbers JF946491–JF946513.

### Recombinant strains

A DNA fragment containing the wild-type *mecI* coding region and the putative ribosomal binding site from the prototype strain N315 was amplified by PCR with the high-fidelity *Pfu* Turbo DNA polymerase (Stratagene, La Jolla, CA) with primers 5′-ATG GGA ATT CAG CAC AAC AAA TTT CTG AGC-3′ (forward) and 5′-AGA GGG GAT CCT CAA CGA CTT GAT TGT TTC C-3′ (reverse) containing the underlined recognition sequences for endonucleases *Eco*RI and *Bam*HI, respectively. Using the same strategy a PCR fragment containing a non-functional *mecI* coding sequence (mecI*) was obtained from strain ANS46, a prototype strain for SCC*mec* type III strain with a non-sense mutation at Gln_68_ in the *mecI* coding sequence [22], [36]. (CAA→TAA) . A DNA fragment containing the wild-type *mecI-mecR1* coding regions and the putative ribosomal binding sites from the prototype strain N315 was also amplified by PCR with *Pfu* Turbo DNA polymerase with forward primer 5′-GTT CGA ATT CTT CTA CTT CAC CAT TAT CGC-3′ (containing the underlined recognition sequences for endonuclease *Eco*RI) and the same reverse primer described above. After double digestion and purification, the inserts were directionally cloned into the multiple cloning site of pGC2. pGC2 is a high-copy number *E. coli-S. aureus* shuttle plasmid with resistance determinants to ampicillin (*E. coli*) and chloramphenicol (*S. aureus*), obtained from P. Matthews, in which the multiple cloning site is flanked by the strong SP6 and T7 bacteriophage promoters. After ligation the recombinant plasmids were transformed and propagated in *E. coli* strain DH5α. The integrity of the *mecI* ^WT^, mecI*, and *mecImecR1* insert sequences was verified by DNA restriction analysis and sequencing. The recombinant plasmids were then introduced into the restriction-deficient *S. aureus* strain RN4220 (R. Novick) by electroporation with a Gene Pulser apparatus (Bio-Rad, Hercules, Calif.) essentially as previously described [37] and then transduced into the MRSA clinical strains by using phage 80α, as previously described [38].

For the electrophoretic mobility shift assay, the wild-type coding sequences of MecI and BlaI were fused to a histidine tag at the N-terminal. Inserts were obtained by PCR with the *Pfu* Turbo DNA polymerase using DNA from the prototype strain N315 as template and the following primers (5′→3′): mecI Q1, TCA GGG ATC CGA TAA TAA AAC GTA TGA AAT ATC ATC TGC (forward); mecI Q2, GAG GAA GCT TTC AAC GAC TTG ATT GTT TCC (reverse); blaI Q1, GTC TGG ATC CGC CAA TAA GCA AGT TGA ATA TCT ATG G-3 (forward); and blaI Q2, GAC AAA GCT TAT TTT CTG TAC ACT CTC ATC (reverse), containing the underlined recognition sequences for endonucleases *Bam*HI (forward) and *Hind*III (reverse). After double digestion and purification, the inserts were directionally cloned into the multiple cloning site of pQE-30 (Qiagen), an *E. coli* expression vector containing the coding sequence for a tag of histidines upstream the *Bam*H1 restriction site. After ligation the recombinant plasmids were transformed and propagated in *E. coli* strain M15 (Qiagen). The integrity of insert sequences was verified by DNA restriction analysis and sequencing.

### Total RNA isolation and Northern blot analysis

Overnight cultures were grown in TSB, supplemented with chloramphenicol (10 µg/ml) when appropriate, and then diluted 1∶50 in fresh TSB. After cells were grown to the mid-log phase (OD_620_∼0.7), they were pelleted and processed with the FastRNA Blue isolation kit (Bio101, QBiogen) in combination with FastPrep FP120 (Bio101-Savant, QBiogen), according to the manufacturer's recommendations. For the analysis of the *mecA* induction profile, after cultures were grown to OD_620_∼0.7, oxacillin at 0.5 µg/ml was added and cultures were incubated for an additional 60 minutes. Samples were taken at 0, 5, 15, 30, and 60 minutes, pelleted and kept on ice until being simultaneously processed with the FastRNA Blue isolation kit. Total RNA (5 µg) was resolved through a 1.2% agarose-0.66 M formaldehyde gel in MOPS (morpholine propanesulfonic acid) running buffer (Sigma). Blotting of RNA onto Hybond N+ membranes (Amersham) was performed with Turboblotter alkaline transfer systems (Schleicher & Schuell). For detection of *mecA* specific transcripts, a DNA probe was constructed by PCR amplification with primers (5′→3′): *mecA* P1, AAA TCG ATG TAA AGG TTG GC and *mecA* P2, GTT CTG CAG TAC CGG ATT TG. After purification the probe was labeled with a Ready To Go labeling kit (Amersham) by using [a-^32^P]dCTP (Amersham) and was hybridized under high-stringency conditions. The blots were subsequently washed and autoradiographed.

### Electrophoretic mobility shift assay

Expression and purification of MecI and BlaI N-terminal histidine tag fusions (His-MecI and His-BlaI, respectively) was performed in native conditions for 200 ml induced cultures using a Ni-NTA matrix (Qiagen), as recommended by the manufacturer. The purification procedure and recombinant protein purity was evaluated by SDS-PAGE analysis. The concentrations of purified His-MecI and His-BlaI proteins were estimated using the BCA Protein Assay Kit (Pierce), as recommended by the manufacturer. For the electrophoretic mobility shift assay we used the chemiluminescent-based DIG Gel Shift Kit (Roche), following the manufacturer's recommendations. As DNA target we used a c.a. 200 bp fragment encompassing the *mecA* promoter and operator sequences from prototype strain COL obtained by PCR amplification with primers (5′→3′): *mecA* PF1, ATA TCG TGA GCA ATG AAC TG (forward) and *mecA* PR1, TAT ATA CCA AAC CCG ACA AC (reverse).

## Results

### Characterization of the prototype MRSA strains

The resistance-level to oxacillin of the prototype MRSA strains listed in Table 1 was checked with the oxacillin E-test. All strains were classified as fully resistant to oxacillin: MIC≥32 µg/ml. The presence of inducible and functional expression of β-lactamase was also checked through the hydrolysis of the chromogenic substrate nitrocefin for overnight cultures grown in the presence or absence of an inducer (oxacillin at sub-MIC concentration of 0.5 µg/ml). All strains in Table 1, except the reference strain COL, were positive for β-lactamase and its expression was found to be inducible in the presence of oxacillin, suggesting a complete and functional *bla* locus (*blaI-blaR1-blaZ*).

The *mecI* sequence was determined for a total of 11 previously characterized strains positive for the *mecI-mecR1 locus* [22]: three classified as ST5, SCC*mec* type II (including strains N315 and BK2464 listed in Table 1) and eight classified as ST239, SCC*mec* type III (including strains HU25 and BK2421 listed in Table 1). A conserved point mutation was found in all SCC*mec* type III strains. The mutation introduced a premature stop codon at Gln_68_ (CAA→TAA) originating a truncated repressor protein (mecI*). Among the SCC*mec* type II strains, a point mutation (GGAG→GGAA) in the *mecI* ribosomal binding site (RBS) was detected in one strain (BK2464). No mutations were found within the *mecI* coding region for the two other SCC*mec* type II strains. A total of 10 previously characterized strains [22], including all strains listed in Table 1, were also sequenced for the *mecA* promoter region. Except for one mutation in the position −5 detected in a ST5, SCC*mec* type II strain, no point mutations were found in the promoter sequence of the *mecA* gene, when compared to the published sequence for the prototype strain N315 [5].

**Figure 1:**
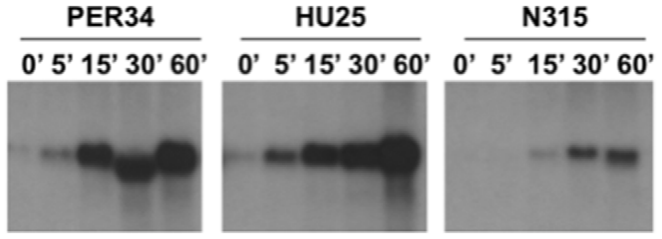
Northern blot analysis of the *mecA* induction profile in three prototype MRSA strains. Induction of *mecA* transcription for three prototype strains upon exposure to oxacillin at 0′, 5′, 15′, 30′, and 60′. Strain relevant characteristics are as follows: strain PER34 - SCC*mec* type I, *mecI* negative, Δ*mecR1*; strain HU25 - SCC*mec* type III, mecI*, *mecR1* ^WT^; strain N315 - SCC*mec* type II, *mecI* ^WT^ *mecR1* ^WT^. The three strains are β-lactamase positive.

The induction profile of *mecA* transcription was checked by northern blotting analysis upon exposure to oxacillin for three prototype strains - PER34, HU25 and N315 - representing different SCC*mec* types and, therefore, with functional and non-functional *mecI-*mecR1 regulatory locus (Table 1 and Figure 1). The three strains tested were shown to have an inducible *mecA* expression independently of being positive or negative for a functional copy of the *mecI-mecR1* locus although there was a remarkable difference in the induction efficiencies: strains PER34 and HU25 (*mecI* negative and truncated *mecI*, respectively) showed a complete induction of *mecA* after 15 minutes, whereas for strain N315 (wild-type sequence for *mecI-mecR1*) the induction was not complete even after 60 minutes of induction. Since the three strains were positive for the β-lactamase locus, these observations confirm that *mecA* transcription can in fact be under the control of the *blaI-blaR1* regulatory *locus* in clinical MRSA strains and are also in agreement with the previous reported differences of induction efficiency of *mecA* between the *mecI-mecR1* and the *blaI-blaR1* systems.

### Overexpression of the *mecA* repressor in prototype MRSA strains

From the above characterization of the prototype MRSA strains listed in Table 1, we were able to justify the high-level oxacillin resistance for strains carrying SCC*mec* types I and III. These were found to have no functional *mecI-mecR1* regulatory loci either due to the characteristic presence of an insertion sequence (SCC*mec* type I) or due to the conserved non-sense mutation within the coding region of *mecI* (SCC*mec* type III) identified in this study. However, for SCC*mec* type II strains we could not explain the high-resistance phenotype since, except for the point mutation in the *mecI* RBS of strain BK2464, no consistent alteration within the *mecI* coding region or *mecA* promoter sequence was detected.

**Figure 2:**
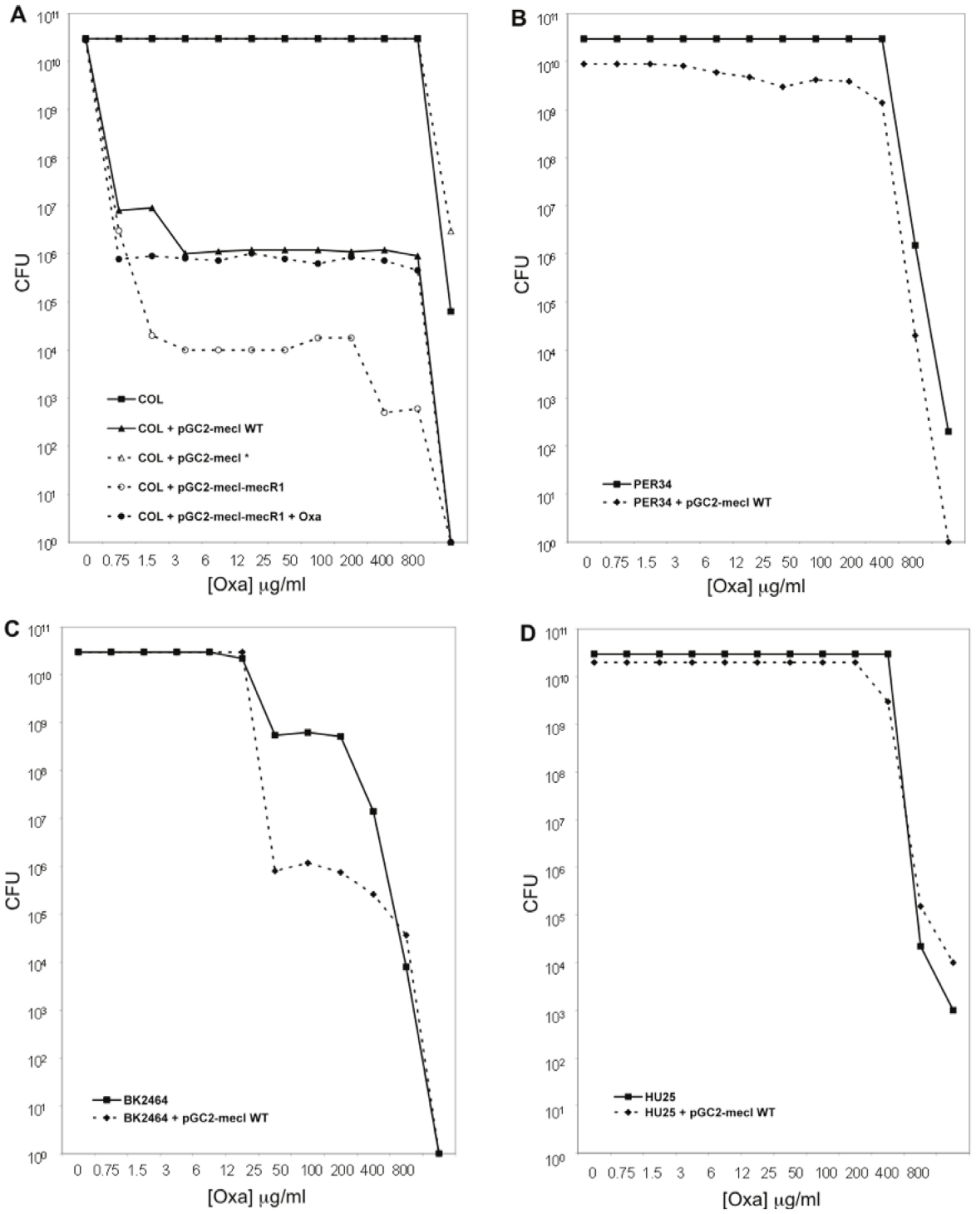
Population analysis profiles of representative parental strains and recombinant strains. Panel A – strain COL and its recombinants strains COL+pGC2-*mecI* ^WT^, COL+pGC2-mecI*, and COL+pGC2-*mecImecR1* grown overnight with and without oxacillin before plating onto TSA plates. Panel B – strain PER34 (SCC*mec* type I) and PER34+pGC2-*mecI* ^WT^. Panel C – strain BK2464 (SCC*mec* type II) and BK2464+pGC2-*mecI* ^WT^. Panel D – strain HU25 (SCC*mec* type III) and HU25+pGC2-*mecI* ^WT^.

To explore these puzzling observations, we have overexpressed *in trans* the *mecA* repressor in the prototype MRSA strains and compared the phenotypic expression of β-lactam resistance between the parental and transformed strains. For this purpose, all strains listed in Table 1 were transformed with a high-copy plasmid containing a wild-type copy of *mecI* of strain N315 (pGC2-*mecI*). Strain COL transformed with pGC2-mecI showed a dramatic decrease of the oxacillin resistance level according to the PAP profile (see Figure 2, panel A) and the MIC as determined by E-test dropped from >256 to 1.5 µg/ml. Strains N315 and BK2464 (SCC*mec* type II) showed a slight decrease of the oxacillin resistance level according to the PAP profiles (see Figure 2, panel C for PAP profile of strain BK2464), although by E-test there was no detectable decrease for strain BK2464 (MIC>256 µg/ml for both parental and recombinant strains) and only a slight decrease for strain N315 (from 32 to 16 µg/ml). Strains PER34 and HPV107 (SCC*mec* type I) and HU25 and BK2421 (SCC*mec* type III) showed no significant alteration of the oxacillin resistance phenotypic expression profile (see Figure 2, panels B and D, for PAP profiles of strains PER34 and HU25, respectively) nor in the MIC as determined by the E-test (MIC>256 µg/ml for all cases).

**Figure 3:**
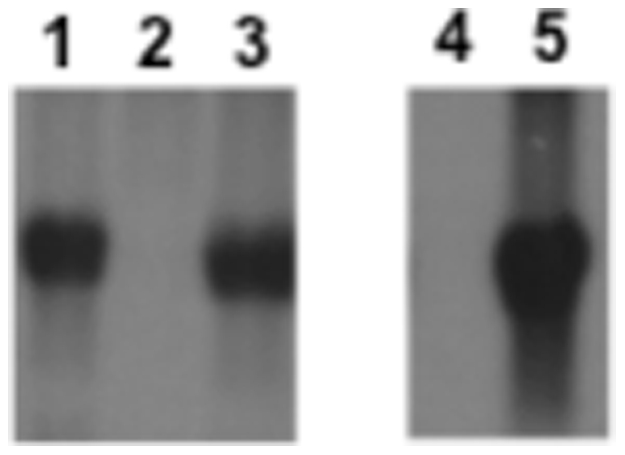
Northern blotting analysis of *mecA* transcription in COL transformants. Lane 1, parental strain COL; lane 2, COL+pGC2-*mecI* ^WT^; lane 3, COL+pGC2-mecI*; lane 4, COL+pGC2-*mecImecR1* uninduced; lane 5, COL+pGC2-*mecImecR1* induced with oxacillin.

A control experiment was done with the mutated *mecI* sequence found in SCC*mec* type III strains (mecI*). The cloning strategy was exactly the same used for obtaining the recombinant plasmid pGC2-*mecI*. When strain COL was transformed with pGC2-mecI* no significant alteration of the oxacillin resistance level was detected – see Figure 2, Panel A. Moreover, the northern blot analysis of the *mecA* transcription showed no differences between COL and COL+pGC2-mecI*, whereas no *mecA* transcript could be detected for COL+pGC2-*mecI* – see Figure 3, lanes 1–3.

Since strain COL is negative for the *mecI* and has a truncated *mecR1*, the phenotype observed for COL+pGC2-*mecI* could be explained by the lack of an inducer of the *mecA* transcription able to release the *mecI*-mediated repression. To test this hypothesis, we have transformed COL with a recombinant plasmid containing the wild-type sequences for both *mecImecR1* (pGC2-*mecImecR1*). Although by northern blotting analysis the *mecA* transcription was restored after overnight growth in the presence of oxacillin (see Figure 3, lanes 4–5), suggesting a fully functional *mecI-mecR1* regulatory system, no significant increase on the oxacillin MIC was detected when compared to COL+pGC2-*mecI*. Moreover, when grown in liquid culture, this recombinant strain exhibited an atypical aggregation of cellular mass in solution (data not shown), suggesting a cellular toxic effect due to the overexpression of MecR1.

### Overexpression of *mecI* in an extended collection of representative MRSA strains

In order to confirm the previous observations, all strains listed in Table 2 were also transformed with the high-copy plasmid containing the wild-type *mecI* (pGC2-*mecI*). This collection includes four additional SCC*mec* type II strains, 13 SCC*mec* type IV strains, and isolates for SCC*mec* types V and VI. SCC*mec* types IV–VI are characterized by deletions in the *mecImecR1* locus due to the presence of insertion sequences. Once again, we could not detect significant alterations in the phenotypic expression of oxacillin resistance upon the overexpression of the *mecA* repressor, except for one strain negative for the nitrocefin assay (VNG17). However, for another nitrocefin negative strain (RJP17) the resistance phenotype was stable. The absence of β-lactamase was confirmed for these two strains by PCR amplification of internal fragments of *blaZ*, *blaI* and *blaR1*. No amplification signals were detected. DNA sequencing of the *mecA* promoter region for both strains revealed no mutations in the operator sequences but a point mutation in the *mecA* ribosomal-binding site was identified by comparison to the wild-type sequence of strain N315 (GGA GGA→GGA GTA).

### Relative affinity of MecI and BlaI for the *mecA* promoter sequence

**Figure 4:**
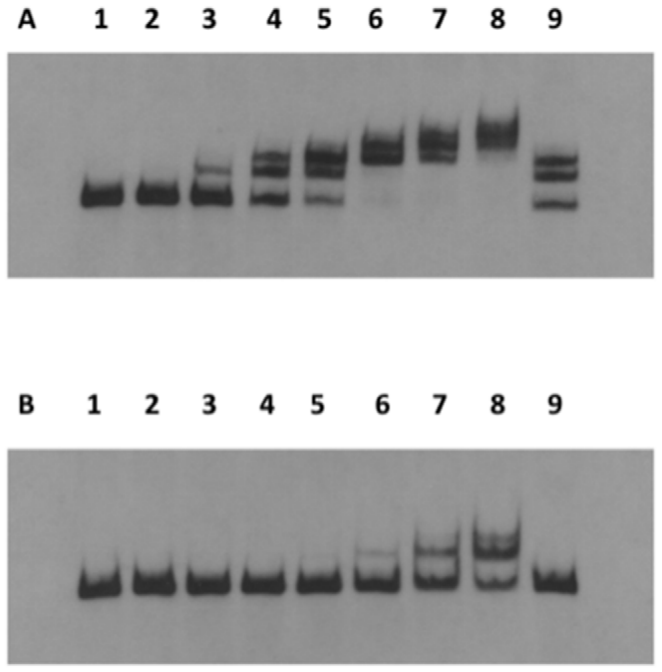
Electrophoretic mobility shift assay of relative MecI and BlaI affinities for the *mecA* promoter sequence. Binding reaction was performed with a labeled 200 bp DNA fragment encompassing the *mecA* promoter mixed with increasing amounts of purified MecI (panel A) and BlaI (panel B). Lanes are as follows: lane 1 – no protein (control); lane 2 – 0,001 µg; lane 3 – 0,01 µg; lane 4 – 0,05 µg; lane 5 – 0,1 µg; lane 6 – 0,25 µg; lane 7 – 0,5 µg; lane 8 – 1 µg; lane 9 – 0,1 ug of protein with a 125 molar excess of unlabelled DNA (specific competition).

In an attempt to explain the lack of effect on the resistance phenotype upon the overexpression of *mecI*, we have evaluated the *in vitro* relative binding affinity of MecI and BlaI repressors to the *mecA* operator sequences by an electrophoretic mobility shift assay (EMSA). For this purpose, we have expressed in *E. coli* N-terminal histidine-tag fusions to the wild-type protein sequences of prototype strain N315 (His-MecI and His-BlaI) and evaluated the binding of purified proteins to a DNA fragment containing the *mecA* promoter sequences of prototype strain COL. As illustrated in Figure 4, by using equivalent concentrations of the purified MecI and BlaI repressors, lower amounts of recombinant MecI protein are required to induce an electrophoretic shift of the DNA fragment containing the *mecA* promoter; i.e., suggesting that the cognate repressor of *mecA* has an increased affinity for its promoter sequences.

## Discussion

The transcription of *mecA*, the gene responsible for the “broad-spectrum” β-lactam resistance in *S. aureus*, may be controlled by two regulatory systems: *mecI-mecR1* and *blaI-blaR1*. Due to the extremely slow induction of *mecA* expression mediated by *mecI-mecR1* regulators, it is believed that the high-level β-lactam resistance characteristic of many contemporary clinical MRSA strains requires a non-functional *mecI-mecR1* regulatory locus [5], [9].

In this study we aimed to understand how the *mecA* transcription is regulated in contemporary pandemic MRSA strains, which in many cases express full β-lactam resistance. Based on previous studies by us and many others on the global epidemiology of MRSA, which included the characterization of the SCC*mec* element, we could, based on the current model of the *mecA* regulation, justify the potential for the expression of high-level β-lactam resistance in many MRSA clones; i.e. all those clones characterized by SCC*mec* types I and IV–VII, which have no-functional *mecI-mecR1* genes due to the presence of insertion sequences. However, for MRSA clones characterized by SCC*mec* types II, III and VIII, which carry a complete *mecI-mecR1* region, we could not explain the high-level β-lactam resistance phenotype. In addition, as illustrated among the MRSA strains selected for this study, no clear relationship exists between SCC*mec* type (i.e. *mecI-mecR1* functionality) and the level of resistance. For instance, low-level resistance is detected among SCC*mec* type IV strains that have no functional *mecI-mecR1* locus.

Upon DNA sequencing of the *mecI* coding region, we found a conserved non-sense mutation at Gln68 in all SCC*mec* type III strains tested. This mutation leads to a truncated MecI protein of 67 a.a. (instead of the 123 a.a. of the wild-type). This mutation appears to be conserved in the ST239-III lineage since it has also been detected in all isolates of a diverse international collection of c.a. 60 ST239-III strains whose genomic sequences have been fully determined [39]. In addition, this very same mutation has been detected in a cluster of 12 clinical MRSA strains and found to be statistically associated to an increase in the *mecA* transcription, suggesting a non-functional MecI protein [40]. In our northern blotting experiments, overexpression of this mutated *mecI* failed to repress the *mecA* transcription on the prototype strain COL that is characterized by a constitutive expression of the resistance determinant. Altogether, these observations suggest that this conserved non-functional version of *mecI* accounts for the high-level resistance phenotype of SCC*mec* type III strains.

Concerning SCC*mec* type II strains, we detected in strain BK2464 a mutation within the ribosomal binding site (RBS) of *mecI*, from GGAG to GGAA. This mutation has also been previously detected among clinical MRSA strains and showed to be statistically associated to a decrease of *mecA* repression [40], which could explain high-levels of β-lactam resistance. However, since we could not detect this mutation in the other two SCC*mec* type II strains analyzed, neither in all genomic and SCC*mec* sequences of type II strains available at GenBank (e.g. strains MRSA252, Mu3, Mu50, JH1, JH9, etc.), it seems that this alteration of the *mecI* RBS *per se* does not justify the β-lactam resistance phenotype of SCC*mec* type II strains. Rosato *et al* have also noticed that the *mecI* RBS differences could not entirely explain the difference in *mecA* transcription between isolates [40]. Regarding the *mecA* promoter, we identified a point mutation found in one single strain in the position −5, which theoretically may affect the binding of MecI [41]. However, this mutation was not detected either in the other SCC*mec* type II strains or in the available genomic sequences and, as such, it also does not justify the resistance phenotype of SCC*mec* type II strains. As we have not sequenced *mecR1*, the hypothesis that the inducer is mutated and able to induce *mecA* transcription more efficiently cannot be excluded. Nevertheless, this hypothesis seems unlikely since no mutations within the *mecR1* coding sequence were detected among genomic sequences available at Genbank, which include SCC*mec* type II and fully oxacillin-resistant strains. In addition, in a large study which addressed the allelic variation of *mecA* regulators only two silent mutations were found within the *mecR1* coding sequence [25].

In order to further explore the puzzling observations described above, we have cloned the wild-type sequence of *mecI* from the reference strain N315 in a high-copy number plasmid with strong bacteriophage promoters flanking the cloning site and we have introduced this recombinant plasmid into prototype and representative MRSA strains. By over-expressing the repressor *in trans*, according to the current model for the *mecA* transcriptional control, we were expecting to see a significant decrease in the resistance level to oxacillin, particularly for strains naturally negative for *mecI* (i.e. containing SCC*mec* types I and IV–VII). To our surprise, except for strains COL and VNG17, the resistant phenotype to oxacillin showed no significant changes in all strains. Since we could see an effect in strains COL and VNG17, we were confident about the functionality of the cloned *mecI*. Nevertheless, we have also transformed COL with the truncated *mecI* found in SCC*mec* type III strains cloned into the same plasmid using exactly the same cloning strategy. As expected, the overexpression of the truncated *mecI* failed to cause any phenotypic alterations. Northern blotting analysis of *mecA* in COL and its transformants also showed that only the wild-type *mecI* was able to repress the *mecA* constitutive transcription of the parental strain. Because COL has no functional *mecR1*, the phenotype observed could be explained by the lack of an inducer able to release the *mecI-*mediated repression on *mecA*, although this would also apply to all other SCC*mec* types I and IV–VI tested for which there was no effect upon the overexpression of *mecI* (except for strain VNG17). Nevertheless, we have transformed COL with a recombinant plasmid containing the full wild-type *mecI-mecR1* system. Interestingly, although we could see induction of *mecA* transcription in the presence of oxacillin, the resistance phenotype of the parental strain could not be restored. Moreover, the transformed strain presented clear physiological perturbations when grown in liquid medium, which suggests that the overexpression of MecR1, a trans-membranar protein, might be toxic for the cell, as previously observed [42].

The two strains for which the *mecI* overexpression caused a decrease in the oxacillin-resistance level (COL and VNG17) were negative for the β-lactamase locus, suggesting that this *locus* might be responsible for the observed “resistance” of MRSA strains to the *mecI* overexpression. Indeed, it has been shown that *blaI-blaR1* can efficiently repress and induce *mecA* transcription [6], [7]; we also confirmed these observations by northern blotting analysis of the *mecA* induction profile in three prototype strains (Figure 1). However, two lines of evidence may reject this hypothesis. First, in another β-lactamase negative strain (RJP17), belonging to the same clone of VNG17 and isolated in the same country and time period, we could not detect significant alterations in the oxacillin resistance phenotype upon the overexpression of *mecI*. Second, although *blaI-blaR1* can efficiently induce *mecA* transcription, the *blaI-blaR1* and *mecI-mecR1* systems are not inter-changeable; i.e. BlaR1 is not able to release the MecI-mediated repression on *mecA* [8]. That is to say, there is no evidence so far supporting the hypothesis that the *blaI-blaR1* system is able to out-compete the MecI-mediated repression on *mecA*, which could eventually account for the lack of effect on the resistance phenotype observed in our overexpression experiments. Moreover, in our experimental system the *blaI-blaR1* expressed from its native promoter would have to out-compete MecI expressed constitutively from a strong bacteriophage promoter.

Inspired by the fact that the *mecI-mecR1* and *blaI-blaR1* systems differ remarkably in the induction efficiency of *mecA*, we have tested the hypothesis that our observations could be explained by differences in the relative affinities of MecI and BlaI for the *mecA* promoter sequences. We have addressed this hypothesis *in vitro* through an electrophoretic mobility shift assay, which clearly showed that lower concentrations of purified MecI are required for detecting a binding to the DNA fragment containing the *mecA* promoter sequences, suggesting an increased affinity when compared to BlaI. Therefore, our observations could not be explained by an increased affinity of BlaR1 for the *mecA* promoter, which eventually would “protect” against the increased cellular amounts of MecI. Actually, this hypothesis would make little sense if one takes into account that the induction process involves the proteolysis of BlaR1 and BlaI and that the sustained expression of the resistance gene requires the continuous expression of both regulatory proteins from their common promoter [43]. In our experimental system, this signal transduction cascade would have to out-compete the binding of MecI (overexpressed constitutively) to the *mecA* promoter.

In short, this study has shown that unexpectedly the β-lactam resistance phenotype of MRSA strains is not affected by the overexpression *in trans* of the *mecA* repressor, even in strains negative for or with non-functional *mecI* gene. This puzzling observation, besides contradicting the current model for the *mecA* transcriptional control in contemporary MRSA strains, strongly suggests that other yet unidentified determinants are involved directly or indirectly in the transcriptional control of the *mecA* gene and, consequently in the phenotypic expression of β-lactam resistance. Elucidation of the nature of these determinants is under way and will be of paramount clinical relevance, since the full understanding of the molecular mechanisms controlling the phenotypic expression of the “broad-spectrum” β-lactam resistance in clinical MRSA strains may contribute to the design of new therapeutic strategies, which may extend the clinical utility of β-lactams.
